# Evaluation of a Targeted COVID-19 Community Outreach Intervention: Case Report for Precision Public Health

**DOI:** 10.2196/47981

**Published:** 2023-12-20

**Authors:** Isela De La Cerda, Cici X Bauer, Kehe Zhang, Miryoung Lee, Michelle Jones, Arturo Rodriguez, Joseph B McCormick, Susan P Fisher-Hoch

**Affiliations:** 1 Department of Epidemiology, Human Genetics and Environmental Sciences School of Public Health Brownsville Campus University of Texas Health Science Center at Houston Brownsville, TX United States; 2 Department of Biostatistics and Data Science School of Public Health University of Texas Health Science Center at Houston Houston, TX United States; 3 Public Health Department City of Brownsville Brownsville, TX United States

**Keywords:** community interventions, emergency preparedness, health disparities, intervention evaluation, precision public health, public health informatics, public health intervention, public health, spatial epidemiology, surveillance

## Abstract

**Background:**

Cameron County, a low-income south Texas-Mexico border county marked by severe health disparities, was consistently among the top counties with the highest COVID-19 mortality in Texas at the onset of the pandemic. The disparity in COVID-19 burden within Texas counties revealed the need for effective interventions to address the specific needs of local health departments and their communities. Publicly available COVID-19 surveillance data were not sufficiently timely or granular to deliver such targeted interventions. An agency-academic collaboration in Cameron used novel geographic information science methods to produce granular COVID-19 surveillance data. These data were used to strategically target an educational outreach intervention named “Boots on the Ground” (BOG) in the City of Brownsville (COB).

**Objective:**

This study aimed to evaluate the impact of a spatially targeted community intervention on daily COVID-19 test counts.

**Methods:**

The agency-academic collaboration between the COB and UTHealth Houston led to the creation of weekly COVID-19 epidemiological reports at the census tract level. These reports guided the selection of census tracts to deliver targeted BOG between April 21 and June 8, 2020. Recordkeeping of the targeted BOG tracts and the intervention dates, along with COVID-19 daily testing counts per census tract, provided data for intervention evaluation. An interrupted time series design was used to evaluate the impact on COVID-19 test counts 2 weeks before and after targeted BOG. A piecewise Poisson regression analysis was used to quantify the slope (sustained) and intercept (immediate) change between pre- and post-BOG COVID-19 daily test count trends. Additional analysis of COB tracts that did not receive targeted BOG was conducted for comparison purposes.

**Results:**

During the intervention period, 18 of the 48 COB census tracts received targeted BOG. Among these, a significant change in the slope between pre- and post-BOG daily test counts was observed in 5 tracts, 80% (n=4) of which had a positive slope change. A positive slope change implied a significant increase in daily COVID-19 test counts 2 weeks after targeted BOG compared to the testing trend observed 2 weeks before intervention. In an additional analysis of the 30 census tracts that did not receive targeted BOG, significant slope changes were observed in 10 tracts, of which positive slope changes were only observed in 20% (n=2). In summary, we found that BOG-targeted tracts had mostly positive daily COVID-19 test count slope changes, whereas untargeted tracts had mostly negative daily COVID-19 test count slope changes.

**Conclusions:**

Evaluation of spatially targeted community interventions is necessary to strengthen the evidence base of this important approach for local emergency preparedness. This report highlights how an academic-agency collaboration established and evaluated the impact of a real-time, targeted intervention delivering precision public health to a small community.

## Introduction

The SARS-CoV-2 pandemic made the dearth of resources and experience to address the situation in local health departments (LHDs) alarmingly apparent. Aggregated data at the state and national levels are insufficiently granular for real-time strategic local interventions. Small communities in particular suffer from limited support and a long turnaround time for obtaining key information needed to conduct effective surveillance and intervention [1]. Texas-Mexico border counties, such as Cameron County, with high burdens of underlying chronic conditions, were heavily impacted by COVID-19 hospitalizations and deaths [2]. By the end of April 2020, Cameron County reported 2.9% case fatalities, exceeding that of its neighboring county, Hidalgo, reporting 0.8%, assuming similarly accurate data for the 2 counties [3]. The LHD of the City of Brownsville (COB), the largest city within Cameron County, accounting for 43% of its total population, recognized specific needs for COVID-19 information dissemination and the resources for assessing community response. The large percentage of the population without insurance, high poverty rates, and elevated rates of type 2 diabetes and obesity (27% and 50%, respectively) contributed substantially to high morbidity and mortality [4,5]. An added obstacle was the cultural and language barriers, given that 85.7% of households speak a language other than English (usually Spanish) and 93.8% are of Hispanic heritage [4]. The COB public health department recognized the limited resources and approached UTHealth Houston School of Public Health, Brownsville (UTHealth), with whom they had a long relationship, to form a partnership to leverage each entity’s strengths to better serve the community [6]. The collaboration began in early April 2020 and had UTHealth faculty and staff helping COB staff conduct COVID-19 case reports and contact tracing tasks. Additionally, weekly sharing of COB in-house COVID-19 case and testing data were accessed by a UTHealth research team to provide census tract-level data and reports to the COB. This team’s innovative acquisition and use of local surveillance data provided the opportunity to strategically target the efforts of the community intervention named “Boots on the Ground” (BOG).

LHDs are the first line of public health action in emergency situations [7]. Their community interventions and actions result in the most immediate response to public health crises. However, resources to effectively implement real-time interventions and assess their impact are rarely available. The “natural experiment” nature of these interventions presents challenges for their evaluation [8]. Experimental methods, such as randomized controlled trials, are considered the gold standard of evaluation. Randomized controlled trials require the random selection of intervention and control groups, which would be costly, impractical, and time-consuming, and raise ethical concerns in regard to respecting community autonomy in crisis situations [9]. Other valuable methods, such as interrupted time series (ITS) studies have been pivotal in health and public policy for evaluating the effects of community-based interventions [10]. ITS studies are used to establish the time series trend of a particular outcome that has been interrupted by an intervention at a specific point in time resulting in self-controlled study units [11]. To our knowledge, there are currently no studies in relation to spatially targeted community interventions for COVID-19 and very few for infectious diseases in general. A 2021 systematic review of spatially targeted community interventions for infectious diseases identified only 10 studies conducted since 1993, none of which properly addressed the evaluation of the intervention [12].

We conducted a study to evaluate the impact of a targeted community COVID-19 education outreach intervention on the SARS-CoV-2 daily test counts by means of an ITS. These findings could help public health professionals and policy makers make authoritative, evidence-based decisions when responding to similar public health crises.

## Methods

### Boots on the Ground

**Figure 1:**
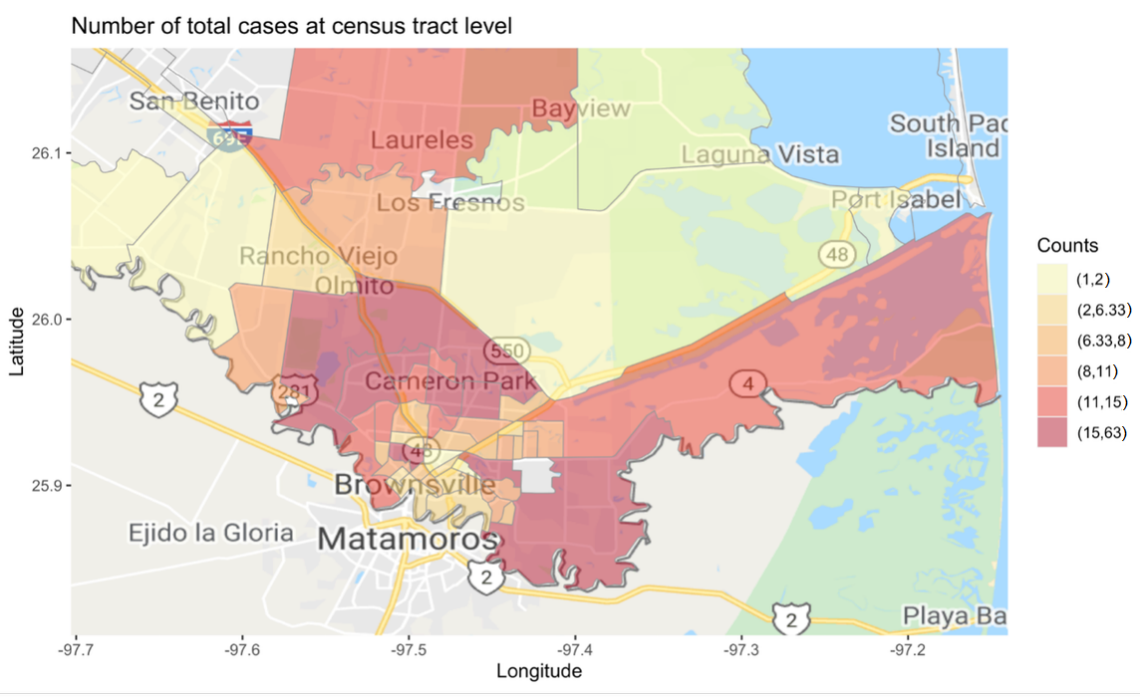
COVID-19 case count maps for City of Brownsville census tracts.

The first official confirmed COVID-19 case report in the COB was on March 21, 2020. The city opened a municipal SARS-CoV-2 testing site and began its BOG efforts to deliver COVID-19 information to COB residents 2 days later [13]. The initial delivery method of BOG was through blanket events opened to all Brownsville residents at the city public libraries. In early April, the COB joined efforts with epidemiological and advanced statistical expertise at UTHealth, which gave rise to the COVID-19 collaborative working group. Locally collected COB COVID-19 case and testing data included geo-codable but otherwise deidentified data and were securely shared among these 2 working groups on a weekly basis. Applying geospatial science methods, the UTHealth partners used these data to deliver granular COVID-19 maps to the COB (48 census tracts). By April 17, 2020, the first COB census tract COVID-19 case maps (Figure 1) were uploaded by the UTHealth team, and on a weekly basis thereafter.

The COB public health department then used these maps to strategically target their BOG efforts at specific census tracts. The COB epidemiologist selected the census tracts with the highest case counts each week for BOG delivery. If census tracts had similar case counts, the tract with higher obesity prevalence was used to determine which tract was selected. The obesity rates were obtained from the 2019 American Community Survey 5-year estimates [14]. A total of 8 bilingual (English and Spanish) COB public health staff members went door-to-door in the streets of selected census tracts, speaking to residents and leaving packets of COVID-19 information at each house. The information packets were provided in English and Spanish and contained educational information on COVID-19 signs and symptoms as well as details regarding free local SARS-CoV-2 polymerase chain reaction testing sites and available testing opportunities. This delivery method reached a total of 17,170 houses in 18 census tracts. Subsequently, on June 23, delivery efforts shifted to broadcasting methods such as radio, social media, vans with loudspeakers, and news outlets.

### Data Sources

The outcome measures chosen to evaluate the impact of BOG were the daily SARS-CoV-2 testing counts. COB COVID-19 testing sites immediately shared all their positive and negative SARS-CoV-2 polymerase chain reaction testing data, which included address information, with the COB. UTHealth experts geocoded addresses to obtain census tract information for each SARS-CoV-2 test, which was then aggregated each day for each of the 18 census tracts. Given the duration of the targeted BOG, from April 21 to June 8, we limited the ITS to only 14 days before and after BOG delivery for each census tract. To avoid a potential spillover effect in our results given the change in BOG delivery method in the last census tract receiving targeted BOG, we limit our time series to only 14 days before and after targeted BOG. For the exposure information, we used the electronic log of BOG efforts maintained by the COB epidemiologist. This log contained the date of targeted BOG delivery for each of the 18 selected census tracts. Based on the log, we created indicator variables representing the pre- and post-BOG day (0 and 1, respectively) status for each tract. An additional variable was created for the time elapsed since BOG delivery for each census tract for the 14 days before and after. We also obtained census tract population size in R Studio’s (R Foundation for Statistical Computing) *tidycensus* package using 2015-2019 American Community Survey data [14,15]. This completed the data necessary to conduct the ITS analysis on each of the census tracts.

### Ethical Considerations

Data user agreements between the COB and UTHealth were approved by city officials. Data privacy protocols were outlined in the agreement. This study was approved by the UTHealth Committee for the Protection of Human Subjects (HSC-SPH-21-1089). This study was exempt from informed consent given that aggregate level and not subject information was being used.

### Statistical Analysis

We hypothesized that the pre- and post-BOG COVID-19 daily test count trend line slope and intercept would significantly increase in BOG-targeted census tracts. For each census tract, we have a series of count data with a known interruption point, which makes piecewise Poisson regression the most suitable model to test our hypothesis [16]. We fit a least squares regression line to each time segment (ie, pre and post) of COVID-19 daily counts, where the change point of the regression line was the day the census tract received targeted BOG, including an offset term of the log of the total population for the census tract. The coefficients of interest in the model were β_2_, which estimates the level or intercept (immediate) change in the number of tests per day after the intervention, and β_3_, which estimates the change in slope (sustained) trend post BOG compared to the pre-BOG trend along with the *P* values of the coefficients. Significance for the coefficients was set at a *P* value of <.05. All statistical analyses were performed in R Studio using the *segmented* package [17,18].

### Additional Analysis

To supplement the interpretation of the 18 census tract results, we conducted an additional analysis. The analysis mentioned above was applied to the 30 census tracts in the COB that did not receive BOG during the targeted BOG phase. For these census tracts, we set a date in the middle of the targeted BOG phase as time 0 (May 18, 2020). This additional analysis provided a comparison group to which we compared our targeted BOG subset analysis results.

## Results

During the targeted phase of BOG, which lasted from April 21 to June 8, 2020, a total of 18 of the 48 census tracts in the COB received BOG outreach, as shown in Figure S1 in Multimedia Appendix 1. To understand the distribution of the daily test counts for each targeted BOG tract, we included Table S1 in Multimedia Appendix 2 for reference. We obtained the 2 parameters of interest (β_2_ and β_3_) and their *P* values for each of the piecewise models of the 18 BOG-targeted census tracts. Using a significance *P* value threshold of <.05, significant intercept change (β_2_) in testing was observed in 2 census tracts, and significant slope (β_3_) change was observed in 5 census tracts. A total of 4 of the 5 tracts with significant slope change had an increase in the slope of the 2-week post-BOG COVID-19 daily test count compared to the pre-BOG slope. A summary of the results is found in Tables 1 and 2.

In the additional analysis of the remaining 30 census tracts in the COB that did not receive BOG outreach during the targeted intervention period, 10 tracts had significant slope changes. A total of 8 of the 10 significant slope changes seen in these census tracts decreased, opposite to what was seen in the slope changes in BOG-targeted census tracts. Table 3 shows the summary of the significant findings and the direction of the coefficients for both groups.

**Table 1:** Piecewise regression coefficients and *P* values for the change of immediate and sustained COVID-19 daily test count trends for 18 census tracts in the City of Brownsville receiving Boots on the Ground outreach.

Census tract	Immediate		Sustained	
	β_2_ coefficient	*P* value	β_3_ coefficient	*P* value
1	0.72	.52	–18.15	.99
2	–0.76	.46	1.55	.61
3	0.005	.96	–0.02	.99
4	4.89	.03	0.45	.28
5	–0.76	.48	–21.44	.99
6	0.04	.96	0.06	.99
7	–0.05	.95	0.43	.21
8	1.14	.11	–0.28	.09
9	0.14	.69	–0.45	<.001
10	–0.45	.62	0.27	.54
11	–0.69	.43	–19.98	.24
12	–2.53	.06	0.77	.83
13	–0.27	.73	0.09	.43
14	1.48	.25	0.48	.11
15	0.07	.92	0.34	.02
16	–1.52	.001	0.18	<.001
17	0.44	.15	0.25	.005
18	0.45	.99	0.10	<.001

**Table 2:** Piecewise regression coefficients and *P* values for the change of immediate and sustained COVID-19 daily test count trends for 30 census tracts in the City of Brownsville not receiving “Boots on the Ground” outreach.

Census tract	Immediate		Sustained	
	β_2_ coefficient	*P* value	β_3_ coefficient	*P* value
1	1.26	.06	0.11	.12
2	0.59	.03	–0.38	.004
3	–1.02	.08	0.18	.54
4	1.32	.04	0.07	.72
5	0.06	.90	–0.43	.57
6	1.28	.01	–0.21	<.001
7	0.17	.80	–18.79	.93
8	0.79	.19	0.37	.80
9	–0.73	.20	0.66	.08
10	0.20	.76	0.15	.16
11	–0.05	.92	–0.17	.99
12	2.50	<.001	–1.36	<.001
13	–0.16	.89	–0.07	.11
14	–0.33	.55	–1.41	<.001
15	1.28	.05	–0.42	.01
16	–0.09	.88	–0.18	.002
17	0.09	.85	0.59	<.001
18	–0.03	.95	–0.95	.47
19	–0.33	.75	0.23	.40
20	1.35	.04	–0.06	.94
21	0.51	.53	1.02	.90
22	2.07	.02	1.17	.08
23	0.13	.86	0.93	.29
24	0.44	.64	0.52	.99
25	–2.83	<.001	–1.79	<.001
26	–0.91	.14	–0.48	.78
27	–0.62	.50	–20.65	.37
28	0.61	.54	0.12	.83
29	–0.72	.25	–1.10	.37
30	0.23	.58	1.94	.002

**Table 3:** Direction of coefficients in the significant trend changes observed.

	Received targeted BOG^a^		Did not receive targeted BOG	
	Immediate (n=2), n	Sustained (n=5), n	Immediate (n=8), n	Sustained (n=10), n
Positive impact	1	4	7	2
Negative impact	1	1	1	8

^a^BOG: Boots on the Ground.

## Discussion

### Principal Findings

The use of local COVID-19 surveillance data, along with collaboration between local public health departments and academia to target a COVID-19 educational outreach intervention, resulted in significant impacts on daily COVID-19 test counts. Most notable was the increase in the sustained trend of testing observed after BOG in census tracts that received targeted BOG. A total of 4 out of 5 census tracts showed significant increased slope changes in testing trends, meaning that the 2-week post-BOG COVID-19 daily test count slope improved significantly from the 2-week pre-BOG slope. Interestingly, the additional analysis of nontargeted BOG COB census tracts was contrary to what we observed in the BOG-targeted tracts. Here, only 20% (2/10) of the census tracts showed a significant increase in sustained testing. This suggests that daily COVID-19 test-seeking in BOG-targeted census tracts saw a sustained improvement (2 weeks after intervention), whereas test-seeking mostly declined in census tracts that did not receive BOG. The perspective of a COB resident documented in an autoethnographic analysis of BOG provides some insight into our interesting findings [6]. The personal method by which targeted BOG census tracts received tailored information potentially reflected a sense of trust among Brownsville residents and made them more receptive to act on the information they were receiving. The long-term benefits of this intervention were observed during the COVID-19 vaccine rollout in early 2021. As BOG delivery shifted later to social media and broadcasting delivery, a city-specific website called BTX Cares was created and served as a trusted source of SARS-CoV2 testing and vaccine information for COB residents [19]. The trust built through the door-to-door delivery of BOG and the use of BTX Cares for COVID-19 information resulted in successful COVID-19 mass vaccination clinic events hosted by the city.

### Limitations

There are some limitations to consider in this study. Although we did account for census tract population size, the short time series evaluated prevented us from establishing or adjusting for temporal or seasonal confounders that might arise in time series analysis. The lack of a control group for comparison is an issue to be considered in this case report. We addressed this limitation by providing a comparison group—those census tracts in the COB that did not receive targeted BOG outreach [20]. It is also possible that selecting census tracts with higher rates of infection also drove testing to some extent, but since the infection levels were not dramatically different between tracts in this small city, this effect is likely to have been limited. Our findings provide grounds for further research in the delivery of translational science through targeted public health.

### Conclusions

The impact on LHDs of COVID-19 in 2020 was extraordinary in its scale, gravity, and range of challenges it presented. Many years of limited public health funding meant that many counties and cities were underresourced, undertrained, and therefore ill-prepared, for the crisis presented by the pandemic. Addressing immediate needs requires collaboration and new technology. Overall, this program was based on the cooperation of the community with local authorities and with the academic center and ultimately resulted in a trusted channel of communication between city residents and public health professionals that persisted during the COVID-19 vaccine rollout. Combining academia and public health institutions was one way to address the many issues. The actual process was in fact inexpensive and relatively simple in overall execution, even though analytic methods were relatively new and sophisticated. This experience was based on a long-term relationship dating back to the H1N1 influenza outbreak, when similarly, a lack of local resources and data were considerable barriers to effective interventions [1]. What we present here is a case for precision public health that uses new technologies to improve local public health efforts by generating tailored and spatially targeted interventions. Our experiences and findings advocate not only for strong agency-academic collaborations but also for improved, granular population surveillance data sources on a national scale that can be leveraged to target interventions and deliver the correct intervention to the high-risk population in a timely manner [21,22].

## Appendix

Multimedia Appendix 1 Map of all Cameron County census tracts. Orange colored census tracts are City of Brownsville (COB) tracts that received targeted Boots on the Ground (BOG). Yellow colored census tracts are COB tracts that did not receive targeted BOG.

Multimedia Appendix 2 24-day SARS-CoV-2 polymerase chain reaction daily test count descriptive statistics for each targeted Boots on the Ground census tract.

## Abbreviations

BOG: Boots on the Ground
COB: City of Brownsville
ITS: interrupted time series
LHD: local health department
UTHealth: UTHealth Houston School of Public Health, Brownsville

## Data Availability

The data sets generated and analyzed during this study are not publicly available due to data user agreements between the City of Brownsville and UTHealth Houston School of Public Health, Brownsville, but are available from the corresponding author on reasonable request.
