# Supplementary material for: Evaluation of a Targeted COVID-19 Community Outreach Intervention: Case Report for Precision Public Health
Source: JMIR Public Health Surveill. 2023 Dec 20;9:e47981. doi: 10.2196/47981 (PMC10765283; doi:10.2196/47981)
Supplement: Multimedia Appendix 1 [file publichealth_v9i1e47981_app1.docx]

**SUPPLEMENTAL FIGURES**


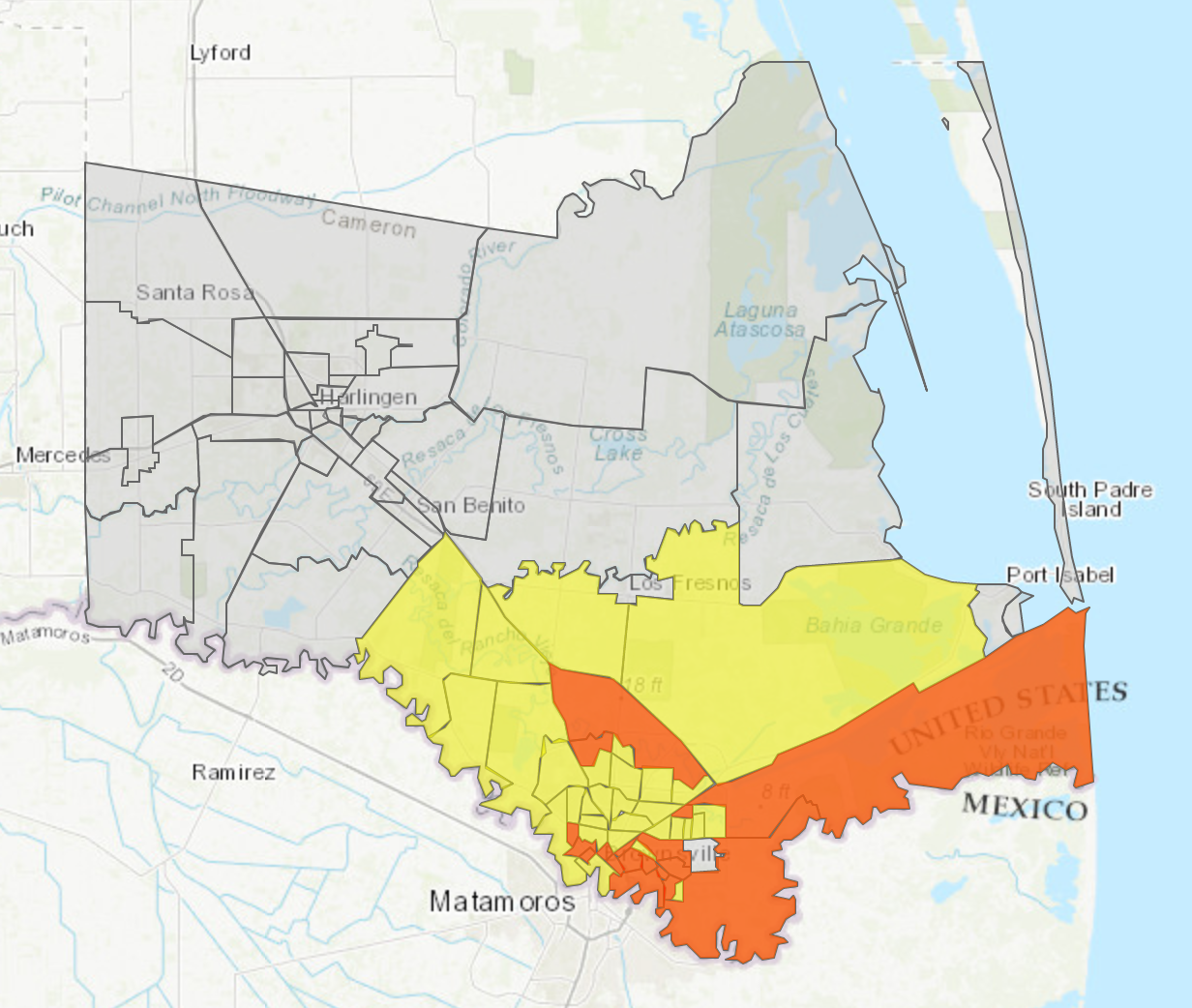


**Figure S1.** Map of all Cameron County census tracts. Orange colored census tracts are COB tracts that received targeted BOG. Yellow colored census tracts are COB tracts that did not receive targeted BOG.
