# Supplementary material for: Evaluation of a Targeted COVID-19 Community Outreach Intervention: Case Report for Precision Public Health
Source: JMIR Public Health Surveill. 2023 Dec 20;9:e47981. doi: 10.2196/47981 (PMC10765283; doi:10.2196/47981)
Supplement: Multimedia Appendix 2 [file publichealth_v9i1e47981_app2.docx]

**SUPPLEMENTAL TABLES**

**Table S1.** 24-day SARS-CoV-2 PCR daily test count descriptive statistics for each targeted BOG census tract.

| Tract # | Total Days without Tests | Total Tests Done | Max Daily Test Count | Minimum Daily Test Count | Population Size |
| --- | --- | --- | --- | --- | --- |
| 1 | 17 | 15 | 3 | 0 | 3978 |
| 2 | 18 | 16 | 4 | 0 | 2593 |
| 3 | 19 | 14 | 4 | 0 | 1758 |
| 4 | 19 | 16 | 4 | 0 | 3549 |
| 5 | 10 | 69 | 10 | 0 | 12498 |
| 6 | 13 | 27 | 4 | 0 | 4708 |
| 7 | 14 | 25 | 4 | 0 | 4577 |
| 8 | 4 | 131 | 62 | 0 | 5021 |
| 9 | 17 | 16 | 3 | 0 | 2096 |
| 10 | 14 | 24 | 4 | 0 | 2311 |
| 11 | 19 | 14 | 3 | 0 | 2431 |
| 12 | 8 | 38 | 5 | 0 | 3190 |
| 13 | 17 | 20 | 3 | 0 | 1999 |
| 14 | 13 | 34 | 5 | 0 | 4042 |
| 15 | 8 | 45 | 5 | 0 | 2839 |
| 16 | 0 | 459 | 64 | 1 | 21039 |
| 17 | 6 | 621 | 79 | 0 | 6364 |
| 18 | 7 | 52 | 3 | 0 | 3204 |
